# Sequential Spinodal Decompositions and Ordering Reactions in an As-Quenched Cr_39_Co_18_Fe_18_Ni_18_Al_7_ High-Entropy Alloy

**DOI:** 10.3390/ma18235364

**Published:** 2025-11-28

**Authors:** Rosemary Chemeli Korir, Gurumayum Robert Kenedy, Wei-Chun Cheng, Shih-Hsun Chen

**Affiliations:** 1 Department of Mechanical Engineering, National Taiwan University of Science and Technology, 43 Keelung Road, Section 4, Taipei 106335, Taiwan; 2 Department of Mechanical Engineering, UPES Dehradun, Energy Acres, P.O. Bidholi, via, Premnagar, Dehradun 248007, Uttarakhand, India; gurumayum.kenedy@ddn.upes.ac.in; 3 Department of Mechanical Engineering, National Yang Ming Chiao Tung University, Hsinchu 300093, Taiwan

**Keywords:** high-entropy alloy, co-clustering effect, ordering effect, spinodal decomposition, ordering reaction

## Abstract

**Highlights:**

**What are the main findings?**

- Sequential spinodal decompositions and ordering reactions are the primary mechanisms of phase transformation
- The BCC phase decomposes into Ni-Al-lean BCC and Ni-Al-enriched BCC phases via spinodal decomposition during cooling
- Ni-Al-enriched BCC phases transform into B2 phases through ordering.
- Two different BCC/B2 microstructures with reciprocal phase distributions were observed.

**What are the implications of the main findings**

- The observed BCC phase transformation pathway aids control of phase evolution in Al–Co–Cr–Fe–Ni HEAs.
- Reciprocal BCC/B2 distribution shows Ni–Al tuning can adjust alloy phase stability and properties.
- The results provide guidance for designing spinodal-strengthened Cr–Co–Fe–Ni–Al HEAs.

**Abstract:**

Constituent phases and their corresponding phase transformations are important in developing alloys. This study investigates the phase transformations of a Cr_39_Co_18_Fe_18_Ni_18_Al_7_ HEA after annealing at and quenching from 1100 °C, 1200 °C and 1300 °C. The as-quenched alloy exhibits major body-centered cubic (BCC) and minor face-centered cubic (FCC) structures. The volume fraction of the BCC phase progressively increases as the annealing temperature is elevated. Upon cooling, the occurrence of spinodal decomposition in the high-temperature BCC phase leads to the formation of two distinct disordered BCC phases, BCC1 and BCC2, at a high temperature regime. The BCC1 phase acts as the matrix and is lean in Ni and Al concentrations, while the BCC2 phase presents as fine particles and is enriched in Ni and Al. As the temperature decreases, sequential spinodal decompositions occur in both BCC phases, giving rise to other product BCC phases. Upon further cooling, the Ni–Al-enriched BCC phases undergo ordering reactions, transforming into B2 phases. Consequently, the major phases in the matrix and fine particles are BCC and B2, respectively. In addition, the BCC matrix and B2 fine particles also contain B2 and BCC nanoparticles, respectively. The co-clustering and ordering effects of Ni and Al participate in the phase transformations of the as-quenched HEA. Correspondingly, the hardness increases with annealing temperature, which is attributed to the higher BCC phase fraction and the increasing number density of ordered B2 precipitates that collectively strengthen the matrix by impeding dislocation motion.

## 1. Introduction

High-entropy alloys (HEAs) based on the Al–Co–Cr–Fe–Ni system exhibit complex phase-stability behavior arising from their multicomponent nature and the interplay of thermodynamic and kinetic factors. Depending on composition and thermal history, these alloys can form FCC, BCC, B2, L1_2_ and σ phases [1,2,3,4,5,6,7,8,9,10,11,12], leading to diverse property combinations including high-temperature strength, corrosion and oxidation resistance, radiation tolerance and excellent cryogenic toughness [1,13,14,15,16,17]. Variations in the alloy compositions and heat treatment have also been shown to modify the constituent phases and corresponding phase transformations, and these changes in turn significantly influence the overall properties of the alloys [3,4,5,6,7,18,19,20,21,22,23,24].

The phase transformations responsible for forming the aforementioned phases in these HEAs mainly include precipitation transformation, spinodal decomposition and/or ordering reactions [4,9,25]. Precipitation transformation occurs when a second phase forms from a supersaturated solid solution serving as the parent phase [26]. This phase transformation process has been reported to result in the formation of phases such as FCC and σ [12,18,27,28] as well as nanoscale L1_2_, B2 and σ phases [4,10,11,17] in Al–Co–Cr–Fe–Ni-based HEAs. Spinodal decomposition, on the other hand, occurs in a multicomponent phase and involves the decomposition of the high-temperature parent phase into two low-temperature product phases with the same crystal structure as their parent phase. This transformation occurs when the alloy composition lies within the miscibility gap, where the free-energy curve exhibits negative curvature and the parent phase becomes unstable to slight composition fluctuations. The process is driven by the reduction in Gibbs free energy as the phase spontaneously separates into regions with slightly different compositions. These fine fluctuations are amplified uniformly throughout the matrix because no nucleation barrier exists. This behavior differs from classical nucleation and growth, which requires overcoming an energy barrier and typically initiates at preferred sites such as grain boundaries or dislocations, resulting in more discrete compositional variations [26,29,30].

Spinodal decomposition has been documented in Al-containing Cr–Co–Fe–Ni systems, where a high-temperature BCC phase separates into compositionally distinct Fe–Cr-enriched BCC and Ni–Al-enriched B2 regions as the alloy cools [9,10,31,32]. These studies establish that decomposition initiates within the BCC phase and that subsequent Ni–Al ordering generates the B2 structure. Moreover, these studies treated the decomposition as a single-step process and did not examine whether the compositionally modified BCC phases formed at high temperatures may themselves become unstable at lower temperatures. This raises the possibility of sequential spinodal events, which could produce hierarchical microstructures, but remain largely unexplored experimentally and insufficiently connected to composition-dependent thermodynamic instability. Consequently, the detailed sequence of phase separation, the interaction between spinodal decomposition and ordering, and their dependence on annealing temperature are still unclear. These gaps motivate a more comprehensive investigation of the transformation pathways in the present alloy. The goal of the present work was to examine the sequential progression of spinodal decompositions and ordering reactions in a Cr_39_Co_18_Fe_18_Ni_18_Al_7_ HEA upon quenching as well as to assess the co-clustering and ordering effects of Ni and Al. The latter were examined because they represent important factors affecting the phase transformations of such alloys.

## 2. Materials and Methods

The HEA examined in this work had the nominal composition Cr_39_Co_18_Fe_18_Ni_18_Al_7_ (at.%). The alloy was melted and cast by vacuum-arc melting a mixture of high-purity Cr, Co, Fe, Ni and Al to obtain a 500 g ingot. The ingot was first homogenized at 1200 °C for 24 h under an argon atmosphere, hot-forged into a plate, then re-homogenized at 1200 °C for another 24 h, followed by water quenching. The resulting plate was sectioned into specimens with dimensions of 10 mm × 10 mm × 2 mm. To investigate temperature-dependent phase stability, the alloy samples were subsequently annealed in a tube furnace at 1100–1300 °C under argon protection to minimize oxidation and suppress volatilization of alloying elements. The samples were held at 1100 °C for 1 h and at 1200 °C or 1300 °C for 30 min before quenching. These temperatures and holding times were selected to ensure that each alloy sample approached its equilibrium high-temperature state prior to quenching. After heat treatment, each sample was mechanically ground to remove approximately 1 mm of the material from both surfaces to eliminate any oxide-containing or chemically altered layers.

Hardness measurements were performed to evaluate the effect of annealing temperature on the mechanical response of the alloy. Samples were ground with silicon carbide papers up to 1500 grit to obtain a smooth, flat surface suitable for indentation. Hardness was measured using a Mitutoyo Rockwell hardness tester (Model HR-400, Mitutoyo Corp., Kanagawa, Japan) on the Rockwell C scale with an applied load of 1471 N and a 5 s dwell time. Five indentations were made at randomly selected locations for each condition, and the average hardness was reported. The error bars shown in the results correspond to the standard error.

Microstructural characterization was conducted using X-ray diffraction (XRD), scanning electron microscopy (SEM), and transmission electron microscopy (TEM). For SEM analysis, samples were mechanically ground using SiC abrasive papers of progressively finer grit (up to 4000 grit), followed by polishing with alumina suspensions down to 0.05 µm to obtain a mirror-like surface. The polished specimens were then etched with a nital solution (2–5 vol.% nitric acid in ethanol) to reveal the microstructure, followed by ultrasonic cleaning in ethanol before observation. For TEM analysis, thin foils were mechanically polished to ~80 µm, punched into 3 mm disks, and electropolished using a twin-jet polisher in a 10 vol.% perchloric acid in ethanol solution at −15 °C.

XRD measurements were conducted using a D2 Phaser diffractometer (Bruker Corporation, Billerica, MA, USA) with Cu Kα radiation (λ = 1.5406 Å) operated at 30 kV and 10 mA. Diffraction patterns were collected over a 2θ range of 20–100° with a step size of 0.02° and a scan rate of 0.1° s^−1^, and phases were identified using DIFFRAC.EVA V4.3 software (Bruker Corporation, Billerica, MA, USA) with the ICDD PDF-2 database.

Morphological features were examined using a Schottky field-emission SEM (JSM-7900F, JEOL Ltd., Tokyo, Japan) operated at 15 kV and 8 mA to characterize surface morphology and microstructural contrast. Higher resolution microstructural and crystallographic analyses were performed using a Talos F200X G2 TEM (Thermo Fisher Scientific, Waltham, MA, USA) operated at 200 kV. Selected-area diffraction patterns (SADP) and dark-field (DF) images were acquired to identify phases and determine crystal structures. Compositional mapping and microchemical characterization were carried out using scanning transmission electron microscopy–energy-dispersive X-ray spectroscopy (STEM–EDS) and quantified with the Super-X EDS system (Thermo Fisher Scientific, Waltham, MA, USA) integrated within the TEM.

## 3. Results

**Figure 1:**
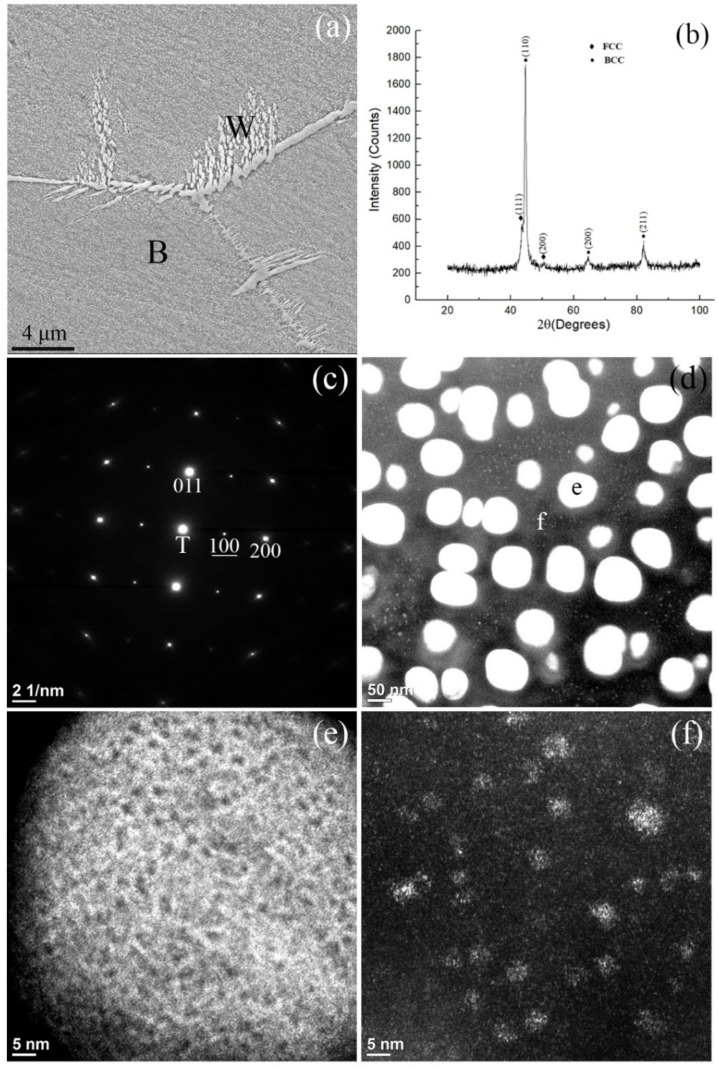
Microstructural characterization of the 1300 °C WQ alloy: (**a**) a secondary electron image (B: BCC, W: Wid. side-plate), (**b**) an XRD pattern (F: FCC), (**c**) the SADP acquired along the [01$\bar{1}$] zone axes of B2 and BCC phases and the Miller index of the B2 reflections indicated with underlining, (T: transmission beam), (**d**) a DF image obtained from the B2 (100) superlattice reflection, (**e**) and (**f**) enlarged DF images acquired from the regions in (**d**) labeled e and f, respectively.

Figure 1 shows the results of microstructural characterizations of the HEA annealed at 1300 °C for 30 min and then subjected to water quench (WQ). This specimen is referred to herein as the 1300 °C WQ alloy and the others are referred to using similar nomenclature as well. The SEM secondary electron image (SEI) provided in Figure 1a indicates that matrix grains, fine particles precipitated homogeneously throughout the matrix, and grain boundary precipitates were present in the form of Widmanstätten (Wid.) side-plates. The XRD pattern in Figure 1b shows that the alloy was composed of a major BCC phase and a minor FCC phase. It is apparent that the major BCC phase is the matrix phase and the minor FCC phase is the grain boundary precipitates. TEM was used to further characterize the crystal structure of the as-quenched alloy and the results are shown in Figure 1c–f. Figure 1c shows an SADP obtained from the matrix grain that exhibits both BCC and B2 reflections. The zone axes of both phases as shown in Figure 1c were captured from the [01$\bar{1}$] directions. The Miller indices of the B2 superlattice reflections are underlined to distinguish them from those of BCC. Detailed TEM images of the B2 and BCC phases are shown in Figure 1d–f.

Figure 1d reveals a dark-field (DF) image obtained from the B2 (100) superlattice reflection shown in Figure 1c. This image demonstrates the uniform distribution of fine oval-shaped particles that have a bright contrast relative to the dark BCC matrix. These fine particles can also be barely seen in the matrix grains in the SEI shown in Figure 1a. These fine particles within the matrix occupied a volume fraction of approximately 50%. Another DF image showing the fine particles but acquired at higher magnification at the fine particle labeled as “e” in Figure 1d is provided in Figure 1e. Interestingly, after adjusting the brightness and contrast of the DF image in Figure 1e, nanoparticles having a dark contrast were found to be uniformly distributed in the fine particle. Because the BCC phase would not be expected to appear in the B2 (100) DF image, it is most likely that these dark nanoparticles comprised a BCC phase. The other magnified DF image is shown in Figure 1f, taken from the matrix region indicated as “f” in Figure 1d. Nano-size particles corresponding to a B2 phase with a bright contrast can also be observed in Figure 1f, but are much smaller than the B2 fine oval-shaped particles in Figure 1d. Thus, the DF images in Figure 1d,f show that the B2 particles were present in the BCC matrix with at least two distinct sizes. The larger particles with diameters ranging from about 50 to 100 nm as seen in Figure 1d are referred to as fine particles, while the smaller particles, with diameters ranging from 2 to 10 nm in Figure 1f, are termed as nanoparticles.

**Figure 2:**
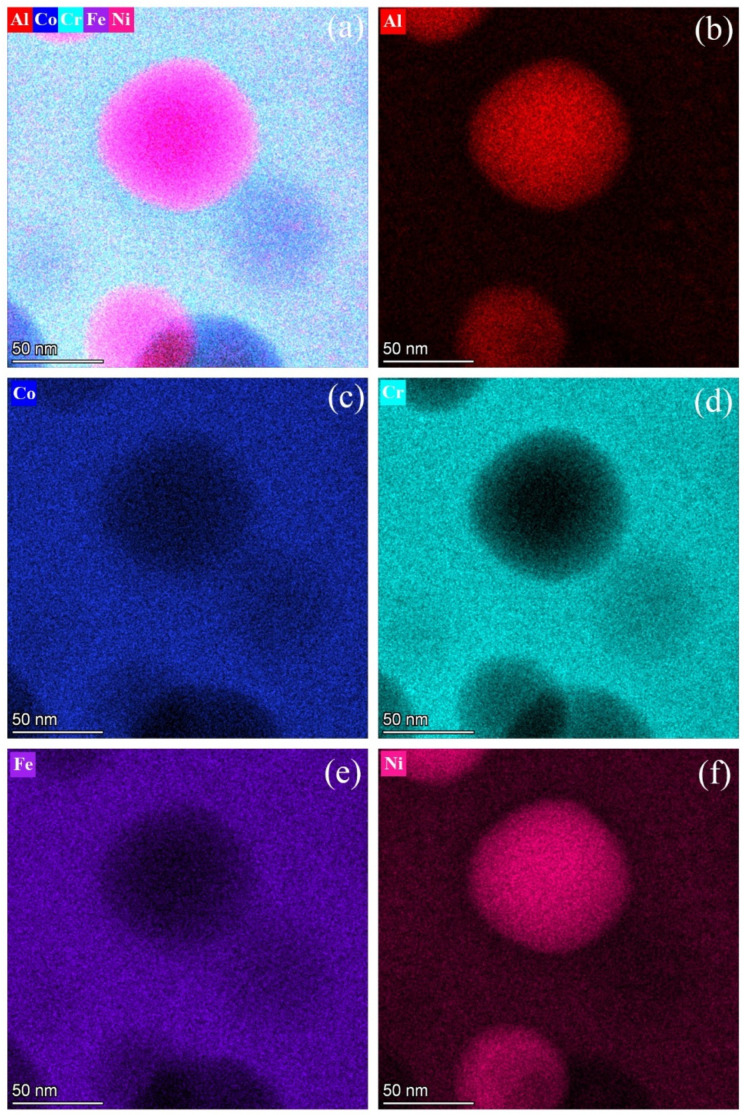
Elemental mappings of the BCC matrix grain of the 1300 °C WQ HEA by the STEM-EDS: (**a**) an overlay mapping; the corresponding elemental mappings for (**b**) Al, (**c**) Co, (**d**) Cr, (**e**) Fe and (**f**) Ni.

**Figure 3:**
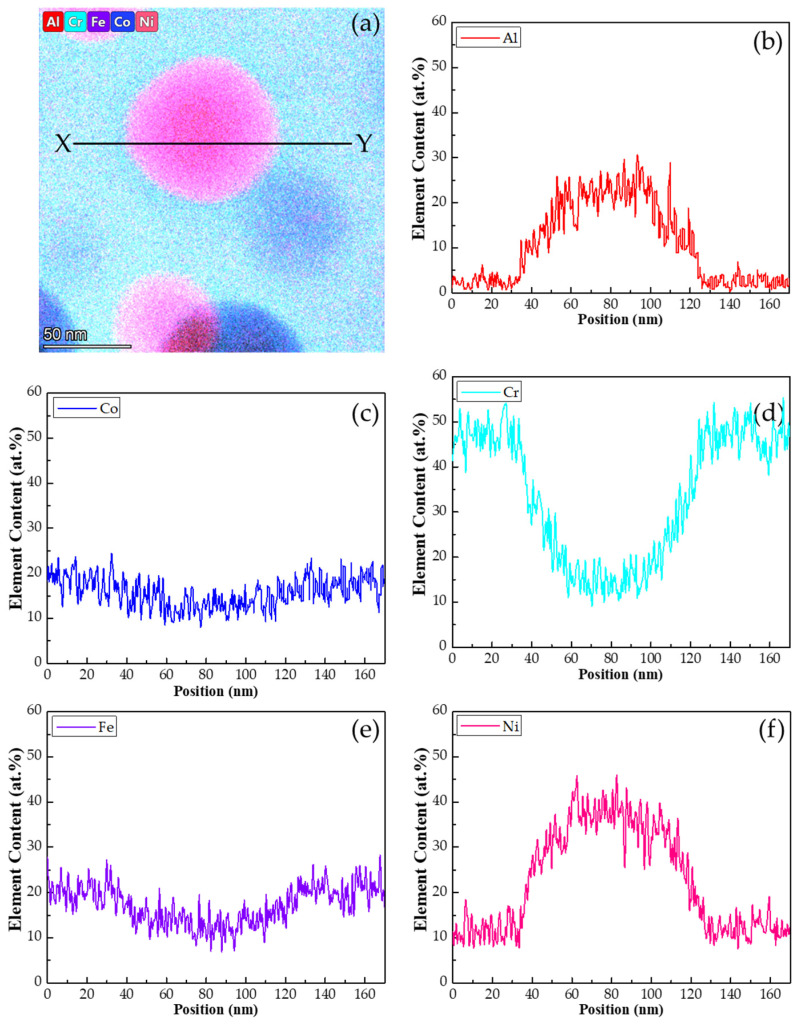
(**a**) An overlay elemental distribution mapping from Figure 2a showing the concentration line scans along the marked line from X to Y and the concentration line scans for various elements as follows: (**b**) Al, (**c**) Co, (**d**) Cr, (**e**) Fe, and (**f**) Ni.

The chemical composition of the constituent phases of the alloy was analyzed using STEM–EDS elemental mapping and line scanning. The mappings presented in Figure 2 reveal the spatial distribution and relative concentrations of the alloy’s constituent elements based on color and intensity, where a brighter intensity indicates a higher concentration of the element. An overlay mapping for all selected elements is shown in Figure 2a, while the individual mappings for Al, Co, Cr, Fe and Ni are shown in Figure 2b–f, respectively. Additionally, STEM line scans, as shown in Figure 3, were taken from the mappings in Figure 2 to demonstrate graphically the distributions of elements along a line which covered both the matrix and the fine particles. The line-scan profiles along the line, XY, in Figure 3a are shown in Figure 3b for Al, 3c for Co, 3d for Cr, 3e for Fe and 3f for Ni. The chemical compositions of the matrix (Cr_49_Co_19_Fe_20_Ni_11_Al_1_) and fine particles (Cr_14_Co_13_Fe_12_Ni_38_Al_23_) were measured using the EDS with an error bar ± 10%. Therefore, the fine particles were enriched in Ni and Al but contained less Co, Cr and Fe, while the opposite was true for the matrix. In previous studies, fine particles reported to comprise a B2 phase were confirmed to have higher concentrations of both Ni and Al [9,10,17,33,34]. Because Al and Ni tend to combine to form a B2 phase [2], the co-clustering and ordering effects of these two elements played important roles in the phase transformations of the HEA during quenching [9,11,13,35].

The EDS technique probed the alloy specimens, including all the composition signals from the surface region to the region with a depth of approximately 10 nm from the surface. Consequently, the spectra obtained from the nanoparticles also included those from their surrounding phases. Thus, the EDS spectra from the nanoparticles could not be accurately acquired because of the nano-sizes of these particles. In addition, the line-scan profiles in Figure 3 indicate significant composition fluctuations of all the selected elements within both the matrix and fine particle. It reveals that the composition fluctuations might result from the composition signals of the neighboring B2 and BCC nanoparticles within the BCC matrix and B2 fine particle, respectively, as shown in Figure 1e,f. In the BCC matrix, the locations of the B2 nanoparticles contain relatively higher Ni and Al concentrations. In the B2 particles, the locations of BCC nanoparticles have relatively lower Ni and Al concentrations. However, from the EDS measurements, the Ni and Al concentrations of the B2 nanoparticles in the BCC matrix are not as high as those of the fine particles as they include the partial concentration signals from the surrounding BCC matrix, and vice versa for the BCC nanoparticles in the B2 fine particles.

The nanoparticles could not be accurately determined by the EDS because of the nano-sizes of these particles. However, the EDS data still provide useful information concerning the compositional distribution trends. As shown in Figure 2b–f and Figure 3b–f, focusing on the fine particle, several locations with sizes similar to those of the dark nanoparticles in Figure 1e can be identified. These regions have low Ni and Al concentrations but higher levels of the other elements than those of the neighboring areas. Therefore, as shown in Figure 1e, the dark nanoparticles (located in the above-mentioned regions) were evidently a BCC phase. In addition, a comparison of Figure 1f with Figure 2 and Figure 3 reveals that the B2 nanoparticles with the brighter contrast in the matrix contained higher levels of Ni and Al but less Co, Cr and Fe. The BCC grains of the HEA quenched from 1300 °C consist of a matrix and fine particles. However, the matrix is a combination of a major BCC phase and a minor B2 phase in the form of the nanoparticles. In contrast, the fine particles comprise a major B2 phase acting as the matrix and a minor BCC phase as the nano-sized particles.

The nanoparticles in either the matrix or the fine particles might have also included smaller nanoparticles of the other phase, as shown in Figure 1e,f. We suggest that the HEA has a single phase of BCC at 1300 °C. The Wid. side-plates of FCC formed in the high-temperature regime during quenching. At the later stage, the fine BCC2 particles form in the BCC1 matrix as the result of spinodal decomposition, that is, BCC → BCC1 + BCC2. Upon further cooling to a lower temperature, separate spinodal decompositions occur in both BCC phases as follows: BCC1 → BCC11 + BCC12, and BCC2 → BCC21 + BCC22. When the temperature drops below the ordering transition temperature (T_o_), the BCC phases with high concentrations of Ni and Al undergo ordering reactions and transform into the B2 phase. Therefore, based on the SADP in Figure 1c, the previous conclusion that B2 superlattice reflections originated only from fine particles while BCC diffraction spots arose solely from the matrix, should be modified [11,36,37].

The entropy effect is an important factor determining the crystal structure of an alloy at high temperatures. Specifically, as the temperature is increased, a more symmetric crystal structure is expected [38]. The BCC phase is more symmetrical than the B2 phase. Therefore, BCC is a high-temperature phase while B2 is a low-temperature phase. According to the phase study shown in Figure 1, Figure 2 and Figure 3, we conclude that the HEA at 1300 °C is a single BCC phase. Upon quenching the HEA from 1300 °C, the FCC phase nucleated along the grain boundaries and grew in the form of Wid. side-plates at the initial stage of cooling, while the high-temperature BCC phase underwent spinodal decomposition, and decomposed into two product disordered BCC phases. These two BCC phases are referred to herein as BCC1 (α1), meaning the matrix phase, and BCC2 (α2), meaning the fine particle phase. Upon further cooling to lower temperatures, these BCC phases also underwent sequential spinodal decompositions, and decomposed into another BCC phase. When the temperature dropped below T_or_, some BCC phases enriched in Ni and Al underwent ordering reactions and transformed into B2 phases.

**Figure 4:**
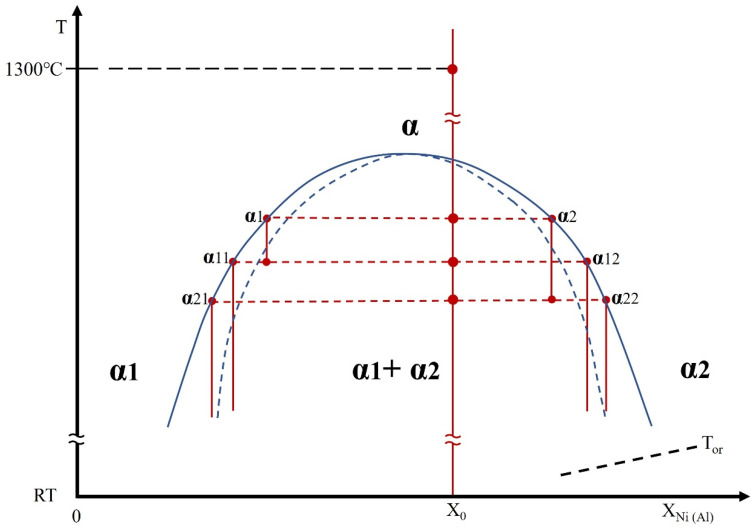
A schematic drawing of a partial phase diagram with a miscibility gap indicating the probable sequential spinodal decompositions and ordering reactions for specific elemental concentrations in the alloy (X: X_Ni_ + X_Al_) (T_or_: ordering transition temperature; RT: room temperature).

The sequential spinodal decomposition observed in this alloy occurs because the Cr–Co–Fe–Ni–Al alloy system passes through several composition-dependent unstable regions within the miscibility gap during cooling. When the alloy first enters the miscibility gap, the high-temperature BCC phase becomes unstable and decomposes into two compositionally distinct low-temperature BCC phases to reduce the Gibbs free energy. As cooling continues, each of these new BCC phases, which have different compositions, subsequently enters its own unstable region, allowing further decomposition. This progression through multiple instability regimes spontaneously results in consecutive spinodal decomposition events and leads to the stepwise phase evolution observed in this study. This understanding of the sequential spinodal decomposition process underpins the schematic representation in Figure 4, which illustrates the consecutive spinodal decompositions and the subsequent ordering reactions that occur during cooling.

Figure 4 shows a schematic drawing of a partial phase diagram containing a miscibility gap. It summarizes the sequential spinodal decompositions and ordering reactions leading to the formation of the BCC and B2 phases, respectively. In this diagram, the product phases ending in 2 (for example, α2, α12, and α22, etc.) are located at the side of the miscibility gap associated with high Ni and Al concentrations but low Co, Cr and Fe concentrations, while the opposite is the case for phases ending in 1. The BCC phases with high concentrations of Ni and Al might have undergone the ordering reaction at temperatures below T_or_ and hence transformed into the B2 phase.

As a result of spinodal decomposition occurring upon quenching the HEA from 1300 °C (i.e., BCC → BCC1 + BCC2), the parent BCC phase (α) existed at 1300 °C and persisted until spinodal decomposition initiated, while the BCC1 (α1) and BCC2 (α2) phases were the resulting spinodal decomposition products. The α1 and α2 phases are located on either side of the miscibility gap, as shown in Figure 4. Thus, in accordance with the TEM observations, it appears that, during cooling of the HEA, both the BCC1 and BCC2 phases also underwent independent spinodal decompositions. These comprised BCC1 (α1) → BCC11 (α11) + BCC12 (α12) and BCC2 (α2) → BCC21 (α21) + BCC22 (22).

The BCC1 (α1) phase initially decomposed into the BCC11 (α11) and BCC12 (α12) phases during quenching. As shown in Figure 4, the α12 phase had high Ni and Al concentrations, while the opposite was true for the α11 phase, and the former phase precipitated homogeneously throughout the α11 matrix in the form of nanoparticles. Upon further cooling, the α 11 and α12 phases could possibly have transformed via other separate spinodal decompositions (i.e., α11 → α111 + α112 and/or α12 → α121 + α122). These BCC phases could thus have transformed through sequential spinodal decompositions. The BCC phases with high concentrations of Ni and Al, such as the α12, α112 and/or α122 phases, might have undergone ordering reactions and transformed, respectively, into nano-sized B2(12), B2(112) and/or B2(122) particles in the α11 matrix. The B2 product phases are thought to have been generated by the ordering reactions of the B2(12), B2(112) and B2(122) phases at temperature below T_or_, allowing them to be distinguished from the B2 phase in the fine particles. However, the B2(12) phase could not co-exist with the B2(122) phase that was obtained from the α122. This implies that the α12 phase underwent spinodal decomposition and not ordering reaction. Evidence for the decomposition of α12 into α121 and α122 and the transformation of α122 into B2(122) in the form of bright contrast spherical nano-sized particles is provided by the smaller dark BCC (α121) nanoparticles seen in Figure 1f. Therefore, the matrix consisted of a major BCC phase and a minor B2 phase in the form of nanoparticles.

The α2 underwent spinodal decomposition during cooling as shown in Figure 4 (meaning α2 → α21 + α22). The resulting α22 phase with high Ni and Al concentrations became the matrix while the α21 was formed as nanoparticles that precipitated homogeneously throughout the fine particles. Upon further cooling, the α21 and α22 possibly further transformed via spinodal decompositions (α21 → α211 + α212 and/or α22 → α221 + α222). Some BCC phases with high Ni and Al concentrations, such as α22, α212 and α222, might have transformed into B2 phases as the temperature dropped below T_or_, which could have resulted in the appearance of B2(22), B2(212) and B2(222) phases. However, the formation of a B2(22) phase from the α22 phase would have been inhibited by the formation of α221 and α222. In contrast, the absence of B2(22) would allow the formation of a B2(222) phase. The existing BCC phases could have continued to decompose via spinodal decompositions with further cooling to room temperature. Therefore, the fine particles made of the BCC2 phase might have undergone a series of phase transformations to produce a crystal structure comprising a major B2 phase as the matrix and a minor BCC phase in the form of nano-sized particles.

**Figure 5:**
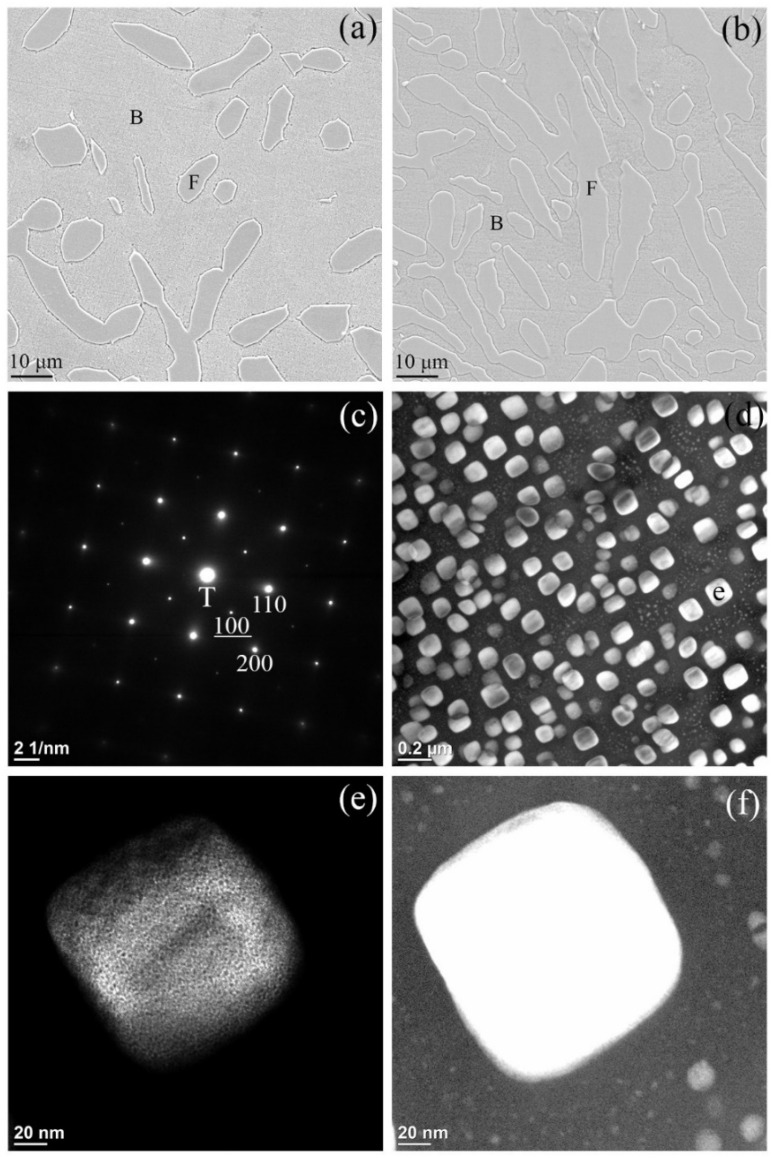
Characterization of the as-quenched HEAs: (**a**) and (**b**) SEIs showing the dual BCC and FCC phases obtained after quenching from 1200 and 1100 °C, respectively, (B: BCC and F: FCC) (**c**) the SADP acquired from the BCC matrix along the [001] zone axes of both the B2 and BCC phases, (**d**) a DF image obtained from the ordered (100) B2 superlattice reflection, (**e**) and (**f**) enlarged DF images with varying brightness and contrast of the same particle labeled as e in (**d**). The images from (**c**) to (**f**) are of the 1200 °C WQ alloy.

Figure 5a,b present SEM micrographs of the 1200 °C WQ and 1100 °C WQ alloy samples, respectively. The SEIs in these figures reveal coexistences of BCC and FCC phases in both as-quenched samples and indicate that both specimens had similar constituent phases. From the combined microstructural observations in Figure 1a and Figure 5a,b, it can be concluded that the HEA within the temperature range of 1100–1200 °C consists of a mixture of BCC and FCC phases. As the temperature decreased, the volume fraction of the FCC phase increased. These results are consistent with the temperature-dependent phase stability as reported in the previous studies [28,39]. At 1300 °C, the alloy is a single BCC phase. When annealed at 1200 °C, some FCC grains form along with the major BCC phase, whereas annealing at 1100 °C results in a near-equal mixture of BCC and FCC phases. This trend aligns with the increasing thermodynamic stability of the FCC phase at lower temperatures.

The SADP acquired from the matrix of the 1200 °C WQ alloy is shown in Figure 5c and exhibits reflections corresponding to both BCC and B2 phases along the [001] direction zone axes, confirming their coexistence. Figure 5d–f present DF images taken from the B2 (100) superlattice reflection in Figure 5c. The DF image in Figure 5d reveals the presence of bright contrast fine cuboidal B2 particles that precipitated homogeneously throughout the dark matrix. Magnified DF images acquired from the fine particle marked as “e” in Figure 5d are displayed in Figure 5e,f. After adjusting the brightness and contrast of the DF image in Figure 5e, additional nanoparticles with a dark contrast distributed uniformly within the fine particles become visible; similar to the analysis of Figure 1e, these nanoparticles are inferred to be BCC phase. Figure 5f presents the same DF image as Figure 5e but with higher brightness to highlight the nanoparticles located adjacent to the fine particle labeled “e” in Figure 5d. These bright-contrast nanoparticles are identified as B2 phase. Overall, the precipitates in the BCC matrix of the 1200 °C WQ alloy exhibit two distinct morphologies and at least two different sizes. Specifically, these were fine cuboidal particles with a width of approximately 100 nm or nano-sized spherical particles with diameters ranging from 2 to 15 nm. The corresponding average chemical compositions of the matrix, fine particles and FCC phase were determined as Cr_52_Co_17_Fe_20_Ni_9_Al_2_, Cr_20_Co_14_Fe_13_Ni_33_Al_20_ and Cr_34_Co_19_Fe_19_Ni_22_Al_6_, respectively.

The hardness of the alloy shows a clear dependence on annealing temperature. The highest hardness was obtained after annealing at 1300 °C (50.42 ± 0.56 HRC), followed by 1200 °C (42.82 ± 0.31 HRC) and 1100 °C (38.96 ± 0.38 HRC). This increasing trend with temperature corresponds well to the microstructural evolution revealed by the samples quenched from each annealing temperature. At higher annealing temperatures, the alloy contains a higher volume fraction of the BCC phase, together with stronger compositional modulation arising from spinodal decomposition and a higher number density of Ni–Al ordered B2 precipitates. These features collectively enhance the resistance to dislocation motion by introducing abundant interphase boundaries, chemical fluctuations and ordered domains, thereby increasing the hardness. In contrast, samples annealed at lower temperatures contain lower volume fraction of BCC phase and less pronounced spinodal and ordering features, resulting in lower hardness values. Similar strengthening contributions from an increased BCC phase fraction and B2 precipitation have been reported in Al-containing Cr–Co–Fe–Ni-based high-entropy alloys [12,35,40].

Each alloy sample was evidently composed of two BCC phases and one FCC phase. Upon quenching, spinodal decompositions occurred within both BCC phases and more BCC phases might have formed sequentially. The BCC phases enriched in Ni and Al could have undergone ordering reactions at low temperatures and generated B2 phases. Therefore, the matrix consisted majorly of a BCC phase while the fine particles were predominantly B2 phase. Nanoparticles were observed within both the BCC matrix and the fine B2 particles in each case these nanoparticles corresponded to the opposite phase.

## 4. Conclusions

This study investigated the phase transformations in a Cr_39_Co_18_Fe_18_Ni_18_Al_7_ high-entropy alloy quenched from 1100 to 1300 °C. The work focused on identifying the constituent phases that form during rapid cooling and the transformation pathways governing their evolution. The results show that the high-temperature BCC phase undergoes spinodal decomposition, followed by ordering at lower temperatures, resulting in a hierarchical microstructure containing BCC and B2 phases. The key findings are summarized below:

i. The alloy quenched from various high temperatures (1100–1300 °C) consists predominantly of BCC and B2 phases with a minor fraction of FCC, and the fraction of BCC increases with higher annealing temperature.
ii. The hardness increases with annealing temperature, aligning well with the increased volume fraction of the BCC phase at higher temperature.
iii. Within the high-temperature BCC grains, BCC and B2 phases form as the final phases in the matrix and in the fine particles, respectively, after quenching.
iv. Nanoparticles consisting of B2 and BCC phases precipitated homogeneously via spinodal decomposition and subsequent ordering reactions in both the matrix and fine particles.
v. A miscibility gap is evidenced by the co-existence of the two BCC phases produced from the spinodal decompositions. The matrix BCC phase, is depleted in Ni and Al and enriched in Co, Cr and Fe, whereas the fine particles in the matrix are enriched in Ni and Al and lean in Co, Cr and Fe.
vi. Upon quenching, the two BCC phases underwent independent spinodal decompositions to produce new product BCC phases that may have further decomposed sequentially into additional BCC phases.
vii. The BCC phases with high Ni and Al concentrations transformed into B2 phases as the temperature decreased below T_or_.
viii. Both the BCC matrix and the fine B2 particles contained nanoparticles consisting of the other phases, formed through sequential spinodal decompositions and ordering reactions that occurred during quenching.
ix. The overall transformation sequence is governed by Ni–Al co-clustering and their strong tendency to order, which promote chemical partitioning and stabilize the resulting phases during cooling. Therefore, the co-clustering and ordering effects of Ni and Al played the dominant roles in controlling the phase transformations of the as-quenched HEA.

## Data Availability

The original contributions presented in this study are included in the article. Further inquiries can be directed to the corresponding authors.

## References

[1] Arshad M., Bano S., Amer M., Janik V., Hayat Q., Bai M. (2024). High-temperature oxidation and phase stability of AlCrCoFeNi high entropy alloy: Insights from in situ HT-XRD and thermodynamic calculations. Materials.

[2] Wang Y., Li B., Ren M., Yang C., Fu H. (2008). Microstructure and compressive properties of AlCrFeCoNi high entropy alloy. Mater. Sci. Eng. A.

[3] Wang W.R., Wang W.L., Wang S.C., Tsai Y.C., Lai C.H., Yeh J.W. (2012). Effects of Al addition on the microstructure and mechanical property of Al_x_CoCrFeNi high-entropy alloys. Intermetallics.

[4] Tong C.J., Chen Y.L., Yeh J.W., Lin S.J., Chen S.K., Shun T.T., Tsau C.H., Chang S.Y. (2005). Microstructure characterization of Al_x_CoCrCuFeNi high-entropy alloy system with multiprincipal elements. Metall. Mater. Trans. A.

[5] Wang W.R., Wang W.L., Yeh J.W. (2014). Phases, microstructure and mechanical properties of Al_x_CoCrFeNi high-entropy alloys at elevated temperatures. J. Alloys Compd..

[6] Kao Y.F., Chen T.J., Chen S.K., Yeh J.W. (2009). Microstructure and mechanical property of as-cast,-homogenized, and-deformed Al_x_CoCrFeNi (0 ≤ x ≤ 2) high-entropy alloys. J. Alloys Compd..

[7] Meshi L., Linden Y., Munitz A., Salhov S., Pinkas M. (2019). Retardation of the σ phase formation in the AlCoCrFeNi multi-component alloy. Mater. Charact..

[8] Shun T.T., Du Y.-C. (2009). Microstructure and tensile behaviors of FCC Al_0.3_CoCrFeNi high entropy alloy. J. Alloys Compd..

[9] Manzoni A., Daoud H., Völkl R., Glatzel U., Wanderka N. (2013). Phase separation in equiatomic AlCoCrFeNi high-entropy alloy. Ultramicroscopy.

[10] Bloomfield M., Christofidou K., Mignanelli P., Reponen A.M., Stone H., Jones N. (2022). Phase stability of the Al_x_CrFeCoNi alloy system. J. Alloys Compd..

[11] Wang Q., Ma Y., Jiang B., Li X., Shi Y., Dong C., Liaw P.K. (2016). A cuboidal B2 nanoprecipitation-enhanced body-centered-cubic alloy Al_0.7_CoCrFe_2_Ni with prominent tensile properties. Scr. Mater..

[12] Joseph J., Stanford N., Hodgson P., Fabijanic D.M. (2017). Understanding the mechanical behaviour and the large strength/ductility differences between FCC and BCC Al_x_CoCrFeNi high entropy alloys. J. Alloys Compd..

[13] Praveen S., Kim H.S. (2018). High-entropy alloys: Potential candidates for high-temperature applications—An overview. Adv. Eng. Mater..

[14] Li L., Lu J., Liu X., Dong T., Zhao X., Yang F., Guo F. (2021). Al_x_CoCrFeNi high entropy alloys with superior hot corrosion resistance to Na_2_SO_4_+ 25% NaCl at 900 °C. Corros. Sci..

[15] Xia S., Yang X., Yang T., Liu S., Zhang Y. (2015). Irradiation resistance in Al_x_CoCrFeNi high entropy alloys. JOM.

[16] Xia S., Gao M., Zhang Y. (2018). Abnormal temperature dependence of impact toughness in Al_x_CoCrFeNi system high entropy alloys. Mater. Chem. Phys..

[17] Tsai M.H., Yeh J.W. (2014). High-entropy alloys: A critical review. Mater. Res. Lett..

[18] Rao J., Diao H., Ocelík V., Vainchtein D., Zhang C., Kuo C., Tang Z., Guo W., Poplawsky J., Zhou Y. (2017). Secondary phases in Al_x_CoCrFeNi high-entropy alloys: An in-situ TEM heating study and thermodynamic appraisal. Acta Mater..

[19] Li C., Li J., Zhao M., Jiang Q. (2010). Effect of aluminum contents on microstructure and properties of Al_x_CoCrFeNi alloys. J. Alloys Compd..

[20] Yang T., Xia S., Liu S., Wang C., Liu S., Zhang Y., Xue J., Yan S., Wang Y. (2015). Effects of AL addition on microstructure and mechanical properties of Al_x_CoCrFeNi High-entropy alloy. Mater. Sci. Eng. A.

[21] Chaudhary V., Gwalani B., Soni V., Ramanujan R., Banerjee R. (2018). Influence of Cr substitution and temperature on hierarchical phase decomposition in the AlCoFeNi high entropy alloy. Sci. Rep..

[22] Santodonato L.J., Zhang Y., Feygenson M., Parish C.M., Gao M.C., Weber R.J., Neuefeind J.C., Tang Z., Liaw P.K. (2015). Deviation from high-entropy configurations in the atomic distributions of a multi-principal-element alloy. Nat. Commun..

[23] Hu J., Li X., Zhao Q., Chen Y., Yang K., Wang Q. (2023). An overview on fatigue of high-entropy alloys. Materials.

[24] Tabassum N., Sistla Y.S. (2025). Thermal stability assessment of mixed phase AlCoCrFeNi high entropy alloy: In silico studies. Phys. Condens. Matter.

[25] Kenedy G.R., Chemeli K.R., Cheng W.C. (2022). The Observation of Cellular Precipitation in an Ni_36_Co_18_Cr_20_Fe_19_Al_7_ High-Entropy Alloy after Quenching and Annealing. Materials.

[26] Porter D.A., Easterling K.E., Sherif M.Y. (2009). Phase Transformations in Metals and Alloys (Revised Reprint).

[27] Strumza E., Hayun S. (2021). Comprehensive study of phase transitions in equiatomic AlCoCrFeNi high-entropy alloy. J. Alloys Compd..

[28] Zhang J., Qiu R., Tan X., Quan X., Song B., Liu Q. (2023). The precipitation behavior in Al_0.3_CoCrFeNi high-entropy alloy affected by deformation and annealing. Metals.

[29] Park H., Haftlang F., Heo Y.U., Seol J.B., Wang Z., Kim H.S. (2024). Periodic spinodal decomposition in double–strengthened medium–entropy alloy. Nat. Commun..

[30] Findik F. (2012). Improvements in spinodal alloys from past to present. Mater. Des..

[31] Bai K., Ng C.K., Lin M., Cheng B., Zeng Y., Wuu D., Lee J.J., Teo S.L., Ng S.R., Tan D.C.C. (2023). Unexpected spinodal decomposition in as-cast eutectic high entropy alloy Al_30_Co_10_Cr_30_Fe_15_Ni_15_. Mater. Des..

[32] Wani I., Bhattacharjee T., Sheikh S., Lu Y., Chatterjee S., Bhattacharjee P.P., Guo S., Tsuji N. (2016). Ultrafine-grained AlCoCrFeNi_2_._1_ eutectic high-entropy alloy. Mater. Res. Lett..

[33] Tsai M.H., Tsai K.Y., Tsai C.W., Lee C., Juan C.C., Yeh J.W. (2013). Criterion for sigma phase formation in Cr-and V-containing high-entropy alloys. Mater. Res. Lett..

[34] Miracle D.B., Senkov O.N. (2017). A critical review of high entropy alloys and related concepts. Acta Mater..

[35] Ma Y., Jiang B., Li C., Wang Q., Dong C., Liaw P.K., Xu F., Sun L. (2017). The BCC/B2 morphologies in Al*_x_*NiCoFeCr high-entropy alloys. Metals.

[36] Borkar T., Chaudhary V., Gwalani B., Choudhuri D., Mikler C.V., Soni V., Alam T., Ramanujan R.V., Banerjee R. (2017). A combinatorial approach for assessing the magnetic properties of high entropy alloys: Role of Cr in AlCo_x_Cr_1–x_FeNi. Adv. Eng. Mater..

[37] Lim K.R., Lee K.S., Lee J.S., Kim J.Y., Chang H.J., Na Y.S. (2017). Dual-phase high-entropy alloys for high-temperature structural applications. J. Alloys Compd..

[38] Filatov S. (2011). General concept of increasing crystal symmetry with an increase in temperature. Cryst. Rep..

[39] Munitz A., Salhov S., Hayun S., Frage N. (2016). Heat treatment impacts the micro-structure and mechanical properties of AlCoCrFeNi high entropy alloy. J. Alloys Compd..

[40] Gupta A., Choudhari A., Rane A., Tiwari A., Sharma P., Gupta A., Sapale P., Tirumala R., Muthaiah R., Kumar A. (2024). Advances in nickel-containing high-entropy alloys: From fundamentals to additive manufacturing. Materials.

